# A Framework for Evaluating AI-Powered Virtual Assistants to Support Older Adults’ Information-Seeking Needs

**DOI:** 10.1093/geroni/igaf122.1640

**Published:** 2025-12-31

**Authors:** Walter Boot, Emily Langston, Varitnan Hattakitjamroen, Mario Hernandez, Hye Soo Lee, Hannah Mason, Willencia Louis-Charles

**Affiliations:** Weill Cornell Medicine, New York, New York, United States; Florida State University, Tallahassee, Florida, United States; University of Illinois Urbana-Champaign, Champaign, Illinois, United States; Weill Cornell Medicine, New York, New York, United States; University of Illinois--Urbana-Champaign, Champaign, Illinois, United States; Weill Cornell Medicine, New York, New York, United States; University of Illinois Urbana-Champaign, Champaign, Illinois, United States

## Abstract

Older adults often face the challenge of searching for critical health, financial, and resource-related information to make complex decisions, a process further complicated by age-related cognitive changes that impact information processing and decision-making. Artificial intelligence (AI)-powered virtual assistants may help by providing concise, easy-to-understand information, yet their accuracy and effectiveness remain unclear. This presentation will introduce a general framework for evaluating AI’s potential to support important decisions of older adults and provide a case example illustrating this approach. To examine the accuracy and utility of AI-powered virtual assistants, we assessed the responses of Alexa, Google Assistant, Bard, and ChatGPT-4 to queries related to Medicare, long-term care insurance, and resource access. Findings showed that Large Language Model (LLM)-based assistants (Bard, ChatGPT-4) were more accurate than non-LLM systems, with Bard producing 6% inaccurate responses compared to Alexa’s 60%. They also provided more supplemental details, with Bard offering high levels of additional information in 79% of responses, compared to 37% for ChatGPT-4 and under 20% for others. However, response variability was observed over time. While LLM-powered virtual assistants may be useful tools for older adults seeking health and financial information, potential inaccuracies, response complexity, and variability must be considered. We will outline key challenges in conducting this research and implementing AI solutions, emphasizing the need for further refinement and user training to enhance reliability and usability for older users.

